# Engaging Patients with Heart Failure in Diet and Nutritional Health Behaviors Through mHealth Applications – A Restricted, Systematic Review

**DOI:** 10.1007/s11897-025-00739-4

**Published:** 2026-02-16

**Authors:** Elisavet Andrikopoulou, Rosalynn C. Austin, Fahad Ahmad, Anne Marie Lunde Husebø

**Affiliations:** 1 School of Computing, PAIDS research centre, Faculty of Technology, University of Portsmouth, Portsmouth, UK; 2 Department of Public Health, Faculty of Health Sciences, University of Stavanger, Stavanger, Norway; 3 Department of Cardiology, Portsmouth Hospitals University NHS Trust, Southwick Hill, Portsmouth, PO6 3LY UK; 4 NIHR Applied Research Collaborative (ARC) Wessex, Innovation Centre, Science Park, 2 Venture Rd, Chilworth, Southampton, SO16 7NP UK; 5 Research Group of Nursing and Health Sciences, Stavanger University Hospital, Stavanger, Norway; 6 School of Computing, Buckingham Building, Lion Terrace, Portsmouth, PO1 3HE UK

**Keywords:** Diet, Heart failure, Health behaviors, MHealth, Nutrition, Patient engagement, Review

## Abstract

**Purpose of Review:**

To examine recent mHealth interventions aimed at supporting diet and nutrition behaviours in heart failure (HF). The review included studies of mobile applications (apps) that incorporated at least one diet- or nutrition-related component published in the last 5 years, in English and targeted for a heart failure population. The review summarises diet and nutrition features and evaluation, engagement strategies within these apps, and reporting of how strategies relate to changes in nutrition-focused health behaviours in people with HF. The review was restricted by period (years) of articles retrieved, percentage of duplication in researchers checking the inclusion and data extraction, and number of databases searched.

**Recent Findings:**

A total of nine studies (2019–2023) met the inclusion criteria. No mHealth application was solely dedicated to diet or nutritional health behaviours in heart failure (HF). Engagement features included personalised feedback, goal setting, reminders, and gamification, which appeared to improve adherence. Ease of use and technical support facilitated patient technology uptake, whereas burdensome data entry and complex interfaces hindered it.

**Summary:**

MHealth apps exist for supporting HF self-care, but only a few include diet and nutrition support. Future app development should integrate robust diet and nutrition guidance alongside standard HF-care. Emphasis on user-centred design, including co-creation with patients and intuitive interfaces is essential to improve usability and engagement. Research is needed to incorporate diet and nutrition management integrate in mHealth tools for those with HF.

**Supplementary Information:**

The online version contains supplementary material available at 10.1007/s11897-025-00739-4.

## Introduction

Heart failure (HF) affects nearly 64 million people worldwide, creating a substantial burden on patients, families, and healthcare systems [1]. HF management involves complex treatments and self-care, putting high demands on the patient. Those demands of adherence with treatment plans and health behaviours is thought to decrease health-related quality of life (HRQoL) [2]. Included in self-care regimens is nutrition and dietary management; including monitoring appetites and food intake (food and vitamins), fluid intake, weight, alcohol consumption [3], however comorbidities such as anxiety and depression may thwart adherence [4]. Patient perceptions about nutrition are crucial to improving dietary adherence in heart failure [5]. Patient education and counselling on diet and other health behaviours (e.g. exercise, keeping up with medications) are important in patients illness management and to avoid deterioration [2]. This support is provided in health appointments but due to the popularity of generic diet and nutrition applications it may be that providing patient led self-monitoring in a mHealth application could provide more supportive care [6].

People with HF are interested in using health apps or connected devices for self-monitoring [7]. In adults with heart disease there is an increasing use of mHealth applications such as wearables and medication trackers to support day-to-day self-management [8]. Existing psychological and practical barriers to dietary adherence are not always fully addressed in routine health appointment, but mHealth applications may help to bridge this gap by delivering tailored education, feedback, and reminders in patients’ everyday environments [9].

Mobile Health (mHealth), defined as ‘the use of mobile and wireless communication technologies to improve healthcare delivery, outcomes, and research’, may efficiently engage patients in heart failure self-care [10, 11]. mHealth applications (apps) may include strategies for health behaviour change in mHealth users. Milne-Ives et al. [12] found that the most used health behaviour changes techniques incorporated in mobile apps were goal setting behaviour (52%), feedback on behaviour (54%), self-monitoring of behaviour (72%), and instructions on how to perform the behaviour (54%) of apps. Strategies such as leveraging affordances, how an application’s features align with users’ capabilities, and using clear signifiers in mHealth design are intended to help patients achieve their health goals. It remains unclear whether such strategies are effectively achieving these goals [13]. While considerable effort is put into the design of human computer interaction mobile applications to secure a straightforward way to use the apps and enable the users to reach their health behaviour goals. More research is needed to establish which health behaviour change techniques provided in mHealth apps increase HF patient engagement in the required self-care of health conditions.

There are two main types of apps: (1) a tethered (tied) app includes features that are not patient-controlled. It can be connected to the data source, including the cloud and the electronic health records or portals, permitting clinician monitoring or input [14]. (2) Untethered mHealth applications mean that the patient-user is the only one permitted to enter, maintain and manage data related to their own health conditions [15]. mHealth apps use different input methods to log dietary information (e.g. text, photo, voice, video inputs) [16]. Regardless of such efforts, mHealth apps may be of acceptable quality but potential for promoting behaviour change is limited.

Although dietary interventions have been explored focusing on how to enhance HF patients’ dietary adherence [5], earlier reviews on HF and nutrition have not yet focused on mHealth solutions [17]. Recent mHealth interventions examine effects on diet quality among mixed long-term illness populations [18–21]. To date, digital interventions involving mHealth applications for patients with HF have mainly focused on medical treatments, symptom monitoring, and health education [22–24]. Healthcare professionals’ clinical capacity is strained, and there is a need for developing more medically informed mHealth interventions to support patients’ adherence to treatment and a healthy lifestyle, inclusive of diet and nutrition [25]. The concept of patient engagement is typically reported as the patients’ adherence to the intervention. Several aspects (e.g., technology usage patterns) of patient engagement in health apps have been identified [26], but limited aspects are supported by healthcare professionals [27]. The lack of supported applications may lead to patients accessing inappropriate guidance and decreasing motivation for using mHealth applications [28]. An overview of the design and strategies to improve HF patients’ engagement in mHealth interventions focused on diet and nutrition choices, and how engagement aligns with improved patient outcomes has not yet been published.

## Aim and Objectives

This restricted, systematic review explores mHealth interventions designed as health behaviour interventions in HF focused on diet and nutrition, and explores how patient engagement with apps might affect health behaviours for patients with HF. The research questions are:

1. Which aspects of diet and nutrition in HF care are reported on in the included studies?
2. What are the user interfaces of the mHealth interventions in terms of usability and interactivity?
3. What are the observed engagement strategies, such as digital design (e.g., gamification) or human components (e.g., phone calls) and any other influence on engagement with mHealth applications?
4. What is the relationship between the engagement strategies and changes in target health-behaviours focusing on diet and nutritional change in the interventions?

## Methods

This study applied a framework for restricted, systematic reviews, as suggested by Pluddemann et al. [29]. Restrictions applied for this review included: number of literature review databases searched and date and language, a percentage of duplicate reviewer screening and verification of data extraction. Restricted reviews are used as a streamlined method useful in fast-moving fields like digital health to provide targeted insights to shape future research developments, hence the inclusion of interventions was restricted to the previous five years to ensure the most up-to-date interventions were captured.

## Literature Search

A Population-Intervention-Comparison-Outcomes-Study Design (PICOS) diagram [30] was developed to aid the decision on search terms (Table 1).

**Table 1:** PICOS element and inclusion and exclusion criteria

PICOS element	Inclusion criteria	Exclusion criteria
Participants	Adults with any kind of heart failure diagnosis Patient treated for heart failure outside of hospital only	Pregnant, cancer, patients terminally ill due to other conditions Children or adolescents with heart failure Inpatients or patients in long-term care facilities
Interventions	Health related mobile applications of any type, focused on diet, nutrition, weight loss/gain, and as a minimum to have a component that includes diet/nutrition	Websites Telephone support only
Comparators	Not applicable	Not applicable
Outcome	Patient engagement strategies Any outcome related to changes in health behaviour with a focus on nutrition (e.g., diet, nutrition, food intake)	Weight monitoring only
Study design type	Original, empirical studies, published in the last 5 years without any geographical restriction	Abstract-only reports, commentaries, commercial studies, party political statements, general discussion papers, magazine or newspaper articles, withdrawn abstracts or articles, protocols of reviews, literature reviews Articles published in other languages than English

PICOS: Population-Intervention-Comparison-Outcomes-Study

A university health librarian (EHM) collaboratively developed the literature search strategy. Search terms covered concepts of mHealth, heart failure and nutrition. Four electronic databases were used for the search: MEDLINE, CINAHL, Web of Science and Scopus. In MEDLINE and CINAHL thesaurus searches were combined with text word searches, while search strategies in Web of Science and Scopus comprised of text word searches in title, abstract and keywords. There was no date restriction in any of the searches. Please see the search strategy in the Supplemental file. The literature search was performed by EHM on April 29, 2024. EHM integrated search results in Endnote v.21 and conducted an automated removal of duplicate titles.

## Study Selection

Returned records were uploaded into Rayyan [31]. Three of the authors (EA, RA, FA) each screened a third of the returned titles and abstracts against the eligibility criteria. One author (AMLH) screened a random sample of titles and abstracts (*n* = 204). The inter-rater reliability [32] was calculated k = 0.86, for the 204 articles with two reviewers which is satisfactory level. Full text review was split between all four authors, and any conflicts were resolved by through discussion by the authors.

## Data Extraction

All authors used a coherent data extraction spreadsheet developed for this study. Data was collected from each included article on study characteristics (i.e., author, publication year, country), study aims, design and methods, engagement strategies, patient adherence, study outcomes.

## Critical Assessment of the Included Studies

Included studies were assessed for methodological soundness using the Mixed Methods Appraisal Tool (MMAT) [33] by the first author, while the last author (AMLH) validated 10% of articles assessed. The overall score was included in the data extraction display, but no studies were excluded based on these scores, aligning with the MMAT developers to the purpose of the this tool [33].

### Data Synthesis

A narrative, interpretive framework [18] was applied in synthesizing the data extracted from the included studies, including the following stages: (1) familiarisation with the data based on the development of a textual description of the studies, (2) thematic analysis, which systematically identified any recurrent themes, and (3) development of a conceptual model to explore relationships in the data. Interventions were grouped using distinct design features and categorised by their scope of use in accordance with a prior systematic literature review and previous health informatics publications [18, 28].

The mhealth design features, outcomes and some engagement strategies (Tables 2, 3 and 4) were informed by prior research on mHealth and user engagement [43, 44]. We also categorised and grouped the engagement strategies based on frequency of use and common elements (Table 5), guided by findings published in a prior scoping review on mHealth engagement strategies [45]. To assess the usability, interactivity and user-friendliness of the included mHealth applications (Table 5) EA retrospectively used Mobile Health App Usability Questionnaire (MAUQ) [46], using data reported in the included articles, with RA independently confirming that evaluation. MAUQ is a validated instrument specifically designed to evaluate mHealth applications, considering the factors such as ease of use, interface design, and user satisfaction. It comprises three tiers namely ease of use, interface and satisfaction and usefulness [46]. The domains of ease of use, interface and satisfaction and usefulness guided our retrospective assessment of user-friendliness and interactivity level into high, medium and low. Finally, the consistency of the results and terminology used was also validated using AI assistance (ChatGPT v. 4).

**Table 2:** Included primary studies characteristics

Study	Study Type	Participants			Intervention	Control	Outcome measures			
		*N*	Mean Age (SD)	Male %	Name	*N*	Patient-Reported Outcome Measures	Behavioral Changes	Heart Failure Outcomes	Findings regarding patient engagement
Bohanec et al. (2021) [34]	RCT	56	63.1 (10.5)	77%	HeartMan DSS	22	SCHFI, MLHFQ, Anxiety & Depression Scales	Adherence to medication reminders, self-care	Dietary advice useful	Patient perceptions on needs for counselling
Carter et al. (2024) [35]	Feasibility Study	14	67.7 (11.7)	43%	BiofourmisRPM	—	SUS, PSSUQ, TAM, USEQ	Daily symptom tracking, BP & weight monitoring	—	Qualitative interviews on usability and acceptability
Choi et al. (2023) [36]	RCT	74	Intervention group: 70.3 (10.5), Control group: 79.4 (7.6)	49%	Heart Failure-Smart Life	38	QoL, HRQoL, Geriatric Depression Scale	Medication adherence (Hill Bone Scale), Self-care	NYHA improvement, LVEF, E/Ea ratio	Engagement with app, interaction with clinicians
Guo et al. (2019) [37]	Quasi-Experimental	66	69.4 (11.2)	52%	HF-based telehealth	—	—	Adherence to HF self-care recommendations	BP, HR, 6MWT trends	Physician-patient engagement
Ismail et al. (2022) [38]	Cross-sectional	902	Median 73 (24–102)	59%	IVR system	—	—	Call completion rate, lifestyle behaviors	—	Readmission associations
Luštrek el al. (2021) [39]	Qualitative	29	Range: 22–39	73%	HeartMan DSS	22	—	Weekly exercise, nutrition, health monitoring	Mostly psychological benefits, and fewer physical benefits.	Motivation to monitor health, patient empowerment, app usability
Nagatomi et al. (2022) [40]	RCT	30	IG: 59.8 (10.0), CG: 67.7 (8.9)	53%	HBCR program	15	KCCQ	Exercise adherence (Fitbit data), diet monitoring	6MWT, BP, Physical function	Interaction with dietitians, adherence to training program
Son et al. (2022) [41]	Quasi-Experimental	100	58.8 (8.8)	83%	Chatbot-based intervention	50	European Heart Failure Self-Care Behavior Scale	Weekly goal setting, self-care scores	—	Gamification impact, patient motivation
Wei et al. (2021) [42]	Pilot RCT	28	Median 63	71%	Habits Heart App	13	Atlanta HF Knowledge Test, KCCQ	Daily to-do lists, diet & symptom tracking	Weight change, sodium tracking	Engagement, duration of app use correlation with outcomes

*N* number of individuals, *SD* standard deviation,* RCT* randomized control trial, *DSS* decision support systems, *RPM* remote patient monitoring, *HF* heart failure, *IVR* interactive voice response, *HBCR* home based cardiac rehabilitation, *KCCQ* Kansas City Cardiomyopathy Questionnaire, *SCHFI* Self-Care Heart FailureIndex, *MLHFQ* Minnesota Living with Heart Failure Questionnaire, *SUS* System Usability Scale, *PSSUQ* Post Study System Usability Questionnaire, *TAM* Technology Assessment Model, *USEQ* Usefulness, Satisfaction, and Ease Questionnaire, *QoL* Quality of Life, *HRQoL* Health Related Quality of Life, *BP* blood pressure, *NYHA* New York Heart Association, *LVEF* left ventricular ejection fraction, *E/Ea* E-wave ration to estimate the left ventricular filling pressures and assess diastolic function *HR* heart rate, *6MWT *6-Minute Walk Test,

**Table 3:** Overview of inclusion of diet and nutrition in the mHealth applications

Paper	Time period	Diet and nutrition tracking	Diet and nutrition assessment	Outcomes
**Bohanec et al.** [34]	3–6 months	Food, liquid and sodium intake	BMI baseline only SCHFI	SCHFI significantly improved self-care maintenance. Patients found the app useful, and the advice about the diet interesting to read. They reported increased knowledge about diets.
**Carter et al.** [35]	30-days	Weight reporting Nutrition education	Community health worker conversation around nutrition	Community health worker interactions were related to reinforcement of salt or nutrition education (*n* = 40, 12.9%)
**Choi et al.** [36]	3 months	Weight reporting Diary of diet status	BMI, WC, EHFScBS	No significant change in BMI and WC. Self-care behaviour changed significantly over time for both control and intervention groups. There was no significant change between groups.
**Guo et al.** [37]	4 months	Weight reporting Reports of low salt, low fat, low sugar consumption Reports of more vegetable consumption	Changes in reported diets/nutrition choices	Increasing numbers of patients reported consuming diets with low salt, low fat and low sugar (B:37, FU:48) and more vegetables (B:22, FU:35)
**Ismail et al.** [38]	12 weeks	Five questions on diet/nutrition: using a saltshaker, eating processed food, drinking eight cups of fluid, reading food labels, eating out safely Two questions around weight: do you weigh yourself daily? Weight gain?	Pattern of responses to the questions asked	IVR positively impacts on HF self-care. Patients reported decreases in reports of “not eating out safely” (17.9% to 7.2%). Age was associated with nutritional behaviors, rural patients were more likely to report not eating processed food and limit fluid intake, and increased readmissions we associated with using saltshakers.
**Lustrek el al. **[39]	3–4 months	BMI, liquid intake, personalized questions on healthy nutrition, eating and drinking behavior	The system assesses the level of patient understanding and provides feedback (positive reinforcement, educational information, and advice on improving and modify their diet.	Patients reported feeling that intervention raised their awareness of lifestyle and health issues. It also was shown to positively affect dietary knowledge (qualitative assessment).
**Nagatomi et al.** [40]	3 months	Weight reporting Meal photos	Geriatric nutritional risk index Controlling nutritional status	There was no significant change in between groups using nutritional questionnaires.
**Son et al.** [41]	6 months	Weight reporting Sodium and water intake	EHFScBS	Statistically significantly higher EHFScBS scores in the intervention group vs. controls. Data not reported on weights, sodium and water intake.
**Wei et al.** [42]	6 weeks	Weight reporting Sodium intake	Count of weight reports and sodium reports	Average of 16 sodium intake logs and 22 weight records. Correlation between application use and weight loss was − 0.4 (*P* = 19). Those in the application group lost weight (3.8lbs/1.7 kg) where those in the control group gained weight (2.5lbs/1.1 kg).

*BMI* body mass index, *SCHFI* Self-Care Heart Failure Index, *N* number of participants, *WC* waist circumference, *EHFScBS* European Heart Failure Self-care Behavior Scale, *B* baseline, *FU* follow up, *IVR* interactive voice response, *HF* heart failure, *P* probability value, Lbs pounds, Kg kilograms

**Table 4:** MHealth applications design features

Study	Intervention	Tethered/Untethered	Connectivity description	User-Friendliness & interactivity level	mHealth features present
Bohanec et al. [34]	HeartMan DSS	Tethered	Connected to external health data sources, providing automatic advice and expert system integration.	High	Self-monitoring, Alerts, Feedback, Personalisation
Carter et al. [35]	BiofourmisRPM	Tethered	Remote patient monitoring (RPM) with community health worker (CHW) guidance.	Medium	Remote monitoring, Education, Messaging, Daily health tracking
Choi et al. [36]	Heart Failure-Smart Life	Tethered	Includes educational materials, chat, and remote access by healthcare providers.	High	Education, Chat function, Self-monitoring, Personalisation
Guo et al. [37]	HCF-based telehealth	Tethered	Integrated web platform, mobile app, and smart devices for patient monitoring and management.	Medium	Data sharing, Remote monitoring, Education, Diary
Ismail et al. [37]	IVR system	Untethered	Automated interactive voice response (IVR) calls for self-monitoring.	Low	Reminders, Automated follow-ups, IVR engagement
Lustrek el al. [39]	HeartMan DSS	Tethered	Connected to cloud services and wearable devices; uses AI-driven decision support for self-care, nutrition, and psychological support.	High	Self-monitoring, Alerts, Feedback, Personalisation, Nutrition advice, psychological support, Wearable integration
Nagatomi et al. [40]	HBCR program	Tethered	Uses Fitbit tracking, clinician tablets, and cloud services.	Medium	Fitness tracking, Remote coaching, Personalised feedback
Son et al. [41]	Chatbot-based intervention	Tethered	Interactive text messaging with integrated cloud services.	High	Gamification, Interactive goal setting, Chatbot communication
Wei et al. [42]	Habits Heart App	Untethered	Patient-controlled daily task tracking with smartphone integration.	Medium	Personalised coaching, Messaging, Daily activity tracking

**Table 5:** Engagement strategies and factors associated with higher adherence in mHealth interventions

Engagement strategy	Description	Adherence factors	Relevant studies
Self-Monitoring & Feedback	Patients track symptoms or behaviours and receive responsive feedback	Higher adherence when feedback was immediate, personalised, and visualised clearly	Bohanec (2021) [34] , Carter (2023) [35], Lustrek (2021) [39]
Educational Content Delivery	Delivery of structured, condition-specific health information	Higher adherence when education was condition-specific, repeated, and supported by clinician follow-up	Choi (2023) [36], Guo (2019) [37], Lustrek (2021) [39]
Reminders & Notifications	Automated prompts to reinforce task completion (e.g. meds, steps, logs)	Higher adherence when reminders were scheduled, consistent, and linked to app-based task tracking	Ismail (2022) [38], Lustrek (2021) [39]
Personalised Feedback	Tailored recommendations based on patient-reported data or behaviour trends	Higher adherence when feedback included motivational framing and practical next steps	Bohanec (2021) [34], Wei (2021) [42], Nagatomi (2022) [40], Lustrek (2021) [39]
Goal Setting	Patient-defined short- and long-term health goals	Higher adherence when goal setting was interactive, clinician-supported, and progress was tracked	Son (2022) [41], Wei (2021) [42], Lustrek (2021) [39]
Gamification	Game-based features (points, levels, rewards) to increase motivation	Higher adherence in short-term use when combined with educational or feedback components	Son (2022) [41], Wei (2021) [42]
Social Support & Interaction	Peer or clinician communication within the intervention	Higher adherence when social features supported emotional wellbeing or when family engagement was present	Son (2022) [41], Nagatomi (2022) [40], Guo (2019) [37]
Integration with Daily Life	Seamless incorporation into user routines (e.g. minimal data entry)	Higher adherence when apps required minimal time and aligned with patient schedules or lifestyle	Ismail (2022) [38], Wei (2021) [42]
Use of Technology & AI	Sensors, wearables, AI-driven decision support or automation	Higher adherence when technology reduced manual input and delivered real-time insight	Carter (2023) [35], Guo (2019) [37], Son (2022) [41], Lustrek (2021) [39]

## Findings

**Fig. 1:**
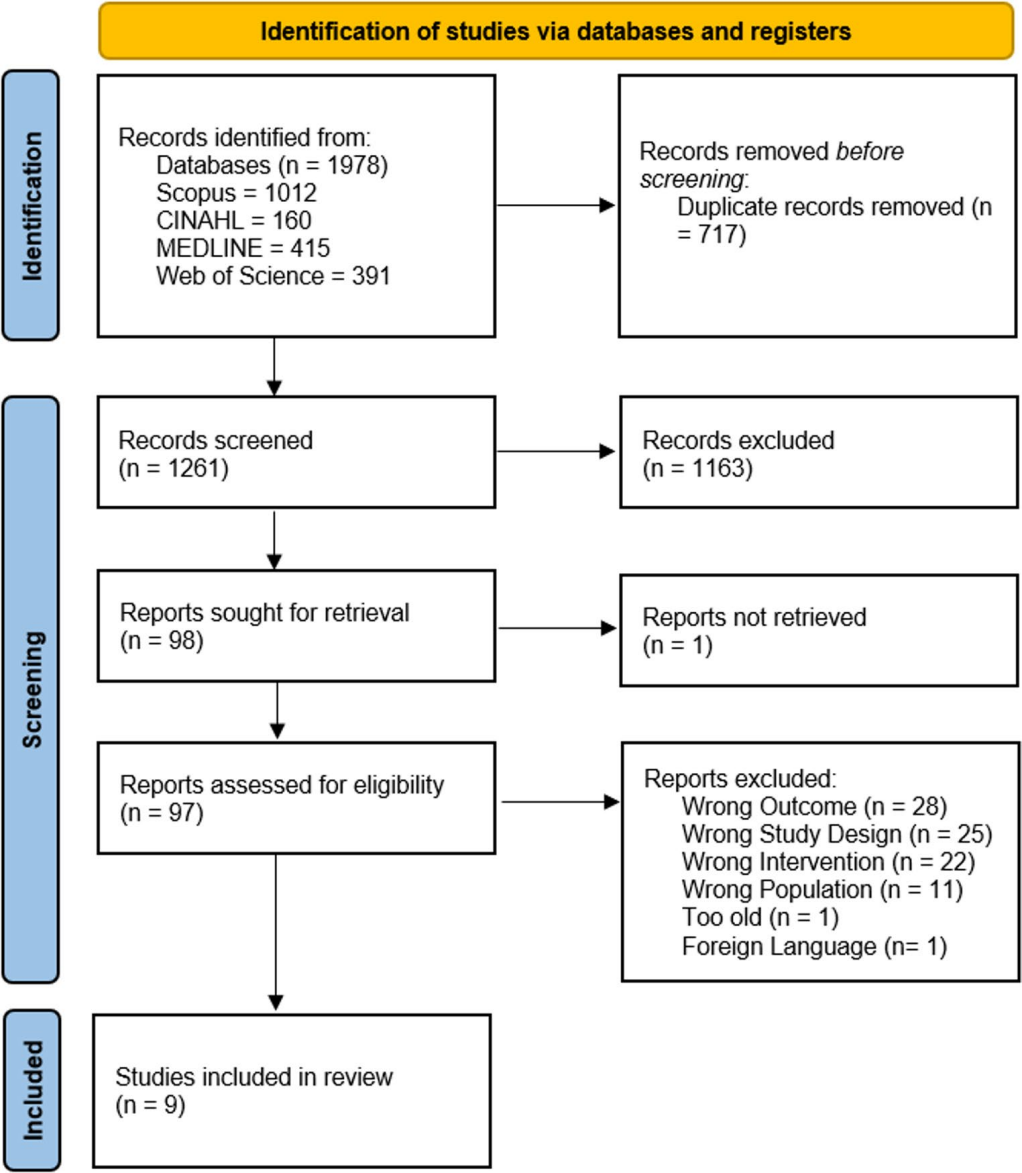
PRISMA literature search and selection method diagram

The search strategies returned 1978 records. After screening titles and abstracts in Rayyan, 197 records remained for full-text assessment. The researcher team decided to apply a further restriction to papers published between 2019 and 2023, and papers in the English language. Leaving a total of 98 articles for retrieval and full text screening. Nine papers were included for full-text assessment. Figure 1 illustrates the literature search and selection method, presenting explanations for the exclusion of studies.

## Study Characteristics

This restricted review included studies where the first authors were from Asia (*n* = 4), North America (*n* = 3), and Europe (*n* = 2). studies were published between 2019 and 2023. Sample size varied from 14 to 100. Most participants were men (> 52%), and ≤ 60 years of age. In Table 2, each study’s design (RCT or observational), intervention features, and key outcomes measured are listed.

Considerable heterogeneity was found across the included studies, as predicted in the protocol [47] in terms of study type (ranging between feasibility studies and RCT’s), intervention type, duration of intervention and follow-up times, and outcomes captured. A meta-analysis was deemed impossible. Instead, a descriptive approach was taken to characterise the studies inclusion and consideration of diet and nutrition in the intervention and in the assessment of the intervention. Data was extracted around three narrative themes: how/if diet/ nutrition was tracked in the application, how diet/nutrition was assessed and any diet/nutrition related outcomes measured.

As a limited number of articles were identified (*n* = 9), we included mHealth applications that purposively incorporated diet or nutrition-supportive components. We employed a Python script supported by ChatGPT-4 to perform a targeted text analysis using Term Frequency–Inverse Document Frequency (TF-IDF) on the abstracts of all included studies [48]. The process involved text extraction, normalisation, and tokenisation, followed by frequency and co-occurrence analysis using predefined keywords for engagement strategies and behaviour change. This enabled us to explore potential associations between strategies (e.g., mobile apps, education) and outcomes (e.g., dietary changes, self-care). The TF-IDF analysis supported our findings, with the highest-ranking terms being *exercise* (0.89), *telehealth* (0.35), *education* (0.27), *depression* (0.09) and *anxiety* (0.09).

### Reports on Diet and Nutritional Health Behaviors

Identified mHealth interventions appeared to be aimed at supporting generic self-care which included a component related to diet and/or nutrition rather than a focused tool (Table 3). Two studies reported on the same mHealth intervention (i.e., HeartMan Decision Support System-DSS) which included app functions (i.e., nutrition questionnaire) to aid in the tracking and reporting of behaviors related to diet or nutrition [34, 36]. The remaining studies relied on patient reports of weight, body mass index (BMI), diet/nutrition questions (i.e., focused on sodium intake, fluid intake, food intake), or patients’ photos of meals.

The assessment of changes diet and nutrition behaviours varied between reporting weight, BMI or waist circumference changes, counts of weight and sodium records, generic questionnaires with at least one question about diet and/or nutrition, question response patterns over time, and topics covered in clinical interactions. One study used two validated tools focused on nutritional status [40]. Two studies developed a list of questions where the patients’ answers would create personalized responses that would include positive reinforcement, educational information, and advice on improving and modifying their diet [34, 39].

Reported outcomes related to change in diet and nutritional behaviors were few and those connected to diet and/or nutrition assessments were again varied (see Table 3). Studies using generic questionnaires did not report separately on nutrition questions [36, 41]. The studies that used diet/nutrition questionnaires reported no significant changes in nutritional status between the control and intervention groups [40].

User interfaces of the mHealth interventions in terms of usability and interactivity.

The application design features identified in this review are presented in Table 4. Every app offered at least self-monitoring or feedback, and many included personalisation. The interventions were differentiated as tethered or untethered (Table 4). For example, the HeartMan system [34, 36] uses wearable sensors and a cloud decision-support platform (tethered), whereas the chatbot app [41] runs independently on patients’ phones (untethered). This distinction highlights how some mHealth tools are integrated with healthcare infrastructure [34, 35, 41], while others operated autonomously. The user friendliness and interactivity retrospective assessment using the MAUQ [46], indicated that all but one study had a medium to high levels of useability (Table 4). The assessment’s rationale, strengths and weaknesses can be found in the supplementary file.

### Engagement Strategies and High Adherence Factors

Table 5 presents the engagement strategies identified in the included studies, their descriptions, and adherence factors (i.e. when higher adherence was observed). Five out of nine strategies showed improved adherence when interventions were interactive, tailored, or incorporated real-time support. Four studies demonstrated that combining multiple strategies, such as education, reminders, and AI-driven personalisation adherence was more effective than single-component approaches.

## Discussion

This review examined mHealth interventions aimed at HF diet and nutritional health behaviors, and explored the relationship between engagement strategies and health outcomes in HF. The lack of studies, focused solely on diet and nutritional health behaviours highlights a gap in mHealth development and an opportunity for future HF apps to incorporate comprehensive nutrition guidance reflecting HF guidelines. The rise of AI tools with the ability to inform nutrition plans, means patients may use these tools, but as highlighted in a recent study, AI generated nutrition plans are not reliable without professional input [49]. Until such tools are developed, clinicians may consider existing generic heart-health or diet apps, but research is needed to validate these for HF patients.

Only one study included a questionnaire developed for the purpose of evaluating diet/nutrition health behaviors [38]. The remaining studies focused assessment on reports of sodium, food, and fluid intake or relied on generic heart failure questionnaires which, at best, included 1–2 questions on diet/nutrition. Unsurprisingly, there is limited ability to conclude on mHealth interventions influence on lifestyle behaviors related to diet/nutrition for patients with HF. With the prevalence of non-illness focused mHealth application on diet/nutrition [50] this represents a missed opportunity to support HF patients in the aspect of their self-care.

Most interventions focused on medication adherence and symptom monitoring, with health behaviors, such as diet included only peripherally. For example, the HeartMan app [34] provided some nutritional advice and activity tracking, and the Habits Heart app [42] enabled healthy habit logging. Consistent with this finding, Thom et al. [51] reported that among 13 mHealth studies on dietary adherence in cardiovascular populations, only one included HF patients. In general, in cardiovascular studies, interactive app features (e.g., text messages or app notifications) have improved diet adherence, but tailored nutrition support for HF remains scarce. No intervention in our review focused solely on dietary counselling.

Usability of the mHealth applications emerged as a key factor in impacting on engagement. Many HF patients are older and may have limited technological experience, so app design must be intuitive. Consistently, studies found that simple, user-friendly interfaces are critical. Wali et al. [52] found that older HF patients would use an app only if it had simple functionality and interface. Common usability issues included burdensome data entry (e.g. logging daily) and lack of integration with electronic records, leading patients to suggest improvements such as streamlining inputs and syncing with health systems. Developers should apply user-centred design: involving patients and caregivers in co-creation can ensure clarity and accessibility of the interface [53].

Even though mHealth applications offer substantial benefits for patients with HF, engagement strategies need further exploration. This review identified that personalised engagement strategies lead to improved patient behaviors, particularly in self-monitoring [34, 35, 37–42, 49, 50]. In contrast, passive strategies like reminders may be less effective over time [35, 36, 38]. This finding is also supported by a recent study exploring the acceptability, usability, and engagement of an mHealth service promoting healthy lifestyle behaviors which found that users appreciated the service’s health focus and felt it gave them a ‘kickstart’ in their behaviour change [54].

Overall, mHealth tools should be designed with user-centred principles, providing clear instructions and minimizing user burden Our findings reinforce that mHealth interventions can be usable for people with HF, but certain user characteristics influence feasibility. Many HF patients are older adults with limited tech experience, and qualitative studies show that unfamiliarity with technology and lack of initial motivation are common challenges in this group [52]. Encouragingly, patients are willing to adopt apps if they are intuitive and well-supported. This is consistent with findings from a systematic review conducted in the context of other chronic conditions (e.g., type 2 diabetes and hypertension) which identified that app-based nutritional programs improved glycaemic control by promoting healthier eating habits and continuous self-monitoring [55].

## Limitations

As highlighted in the methods this review had multiple limitations which aligned with the framework of a restricted review [29]. Limiting to articles published in the last five years may have reduced the number of applications identified. However, due to the pace of application development the focus was on identifying the more recent mHealth applications. The inclusion of only English-language studies may have introduced publication bias, potentially omitting relevant research from non-English-speaking regions. While the original goal of this paper was to describe the presence of diet/nutrition in mHealth applications for people with HF, the heterogeneity in study methodologies and outcome measures made it difficult to compare findings systematically and impossible to conduct a meta-analysis. An iterative change to the methodology, not described in the protocol [47] enabled the description of the assessment and outcomes related to diet/nutrition. Similarly, there was insufficient evidence to summarise usability and engagement specific to diet/nutrition application features, again an iterative focus was broadened to the applications in general. The summarisations should only be used as informative to guide future application refinement and development as the small number of studies included and the heterogeneity between the studies prevents any generalisation.

## Conclusion

Few mHealth applications were identified that support diet and nutritional health behaviors purposely designed for HF patients. Patients with HF as adjuncts to care, thus the current exclusion in HF self-care applications of diet and nutrition health behaviours highlight the opportunity for future app development. It is essential to ensure mHealth applications’ usability and user engagement through focus on user-centred design, and co-creation with end-users. To ensure supportive digital tools to aid in the comprehensive management of HF more research is needed around the inclusion and assessment of dietary and nutritional health behaviours.

## Key References

- Jaarsma T, Hill L, Bayes-Genis A, La Rocca HPB, Castiello T, Čelutkienė J, et al. Self-care of heart failure patients: practical management recommendations from the Heart Failure Association of the European Society of Cardiology. Eur J Heart Fail. 2021;23 [1]:157–74.
  - ○ Outlines the evidence base for self-care in heart failure patients giving guidance across multiple health behaviours for clinicians and patients.
- Forsyth F, Mulrennan S, Burt J, Hartley P, Kuhn I, Lin H, et al. What are the outcomes of dietary interventions in Heart Failure with preserved Ejection Fraction? A systematic review and meta-analysis. European Journal of Cardiovascular Nursing [Internet]. 2023 Oct 1;22 [7]:679–89. Available from: 10.1093/eurjcn/zvac114
  - ○ Review highlighting the dietary and nutrition interventions and their outcomes focused on those with heart failure with preserved ejection fraction.
- Plüddemann A, Aronson JK, Onakpoya I, Heneghan C, Mahtani KR. Redefining rapid reviews: A flexible framework for restricted systematic reviews. BMJ Evid Based Med. 2018;23 [6]:201–3.
  - ○ Framework for the reporting and design of restricted systematic reviews.
- Andrikopoulou E, Austin RC, Fahad A, Husebø MLA. mHealth Interventions to Change Dietary Behaviours in Patients with Heart Failure – Study Protocol of a Restricted Review. In: ITAIS 2024 Proceedings [Internet]. AIS Electronic Library (AISeL); 2024. p. 12–23. Available from: https://aisel.aisnet.org/itais2024
  - ○ The methodological protocol of the current systematic literature review.

## Supplementary Information

Below is the link to the electronic supplementary material.

Supplementary Material 1 (DOCX 25.9 KB)

## Data Availability

No datasets were generated or analysed during the current study.
