# Supplementary material for: Engaging Patients with Heart Failure in Diet and Nutritional Health Behaviors Through mHealth Applications – A Restricted, Systematic Review
Source: Curr Heart Fail Rep. 2026 Feb 16;23(1):8. doi: 10.1007/s11897-025-00739-4 (PMC12909509; doi:10.1007/s11897-025-00739-4)
Supplement: Supplementary file 1 — Supplementary Material 1 (DOCX 25.9 KB) [file 11897_2025_739_MOESM1_ESM.docx]

# User-Friendliness, interactivity, and usability assessment *Rationale*

| Study | User-Friendliness & Interactivity Level | Assessment’s Rationale | Strengths | Weaknesses |
| --- | --- | --- | --- | --- |
| Bohanec (2021) | High | The HeartMan DSS integrates automated advice and expert systems, enhancing system usefulness and interactivity. | Provides personalized feedback and comprehensive monitoring. | May require users to have a certain level of technical proficiency. |
| Carter et al. (2023) | Medium | Biofourmis RPM offers remote monitoring with CHW guidance, improving system usefulness but potentially hindering interface quality due to sensor reliance. | Combines technology with human support for comprehensive care. | Dependence on wearable sensors might pose usability challenges for some users. |
| Choi et al. (2023) | High | Heart Failure-Smart Life provides educational materials and chat functions, enhancing information and interface quality. | Facilitates direct communication with healthcare providers, enriching user experience. | Requires consistent user engagement to maximise benefits. |
| Guo et al. (2019) | Medium | The HCF-based telehealth system integrates various platforms, which may affect interface quality despite high system usefulness. | Offers a multifaceted approach to patient monitoring and management. | Complex integration could lead to a steep learning curve for users. |
| Ismail et al. (2022) | Low | The IVR system focuses on automated calls, limiting interactivity and potentially affecting information quality. | Simplifies self-monitoring through automation. | Lacks real-time engagement, which may reduce user satisfaction. |
| Lustrek et al. (2021) | High | Combines personalized advice, continuous monitoring, and psychological feedback using wearables and cloud AI. | Human-centred design; strong user engagement; includes mental health modules | Physical benefits were less significant; requires wearable device and onboarding |
| Nagatomi et al. (2022) | Medium | The HBCR program uses Fitbit tracking, which may enhance system usefulness but requires technical literacy, affecting interface quality. | Combines fitness tracking with professional coaching for personalized care. | Users with limited technical skills might face challenges in device integration. |
| Son et al. (2022) | High | The chatbot-based intervention incorporates gamification and interactive goal setting, boosting interactivity and interface quality. | Engages users through interactive and motivational features. | Over-reliance on gamification might not suit all user demographics. |
| Wei et al. (2021) | Medium | Habits Heart App offers personalized coaching and daily tracking, enhancing system usefulness but facing usability challenges in interface quality. | Encourages proactive health management through personalized features. | Usability issues could hinder user engagement and satisfaction. |

# Search strategy

We searched Cinahl via Ebsco, Medline via Ovid, Web of Science Core Collection, and Scopus, on April 29, 2024. The search strategies included search terms related to the concepts mHealth, heart failure and nutrition/body weight. In Medline and Cinahl thesaurus searches was combined with textword searches. The Web of Science and Scopus search strategies consist of textword searches in title, abstract and keywords. In Medline, Cinahl and Scopus we removed records containing index terms for animals and not humans. All results were limited to publication years from 2014 to 2024.

Syntax

Proximity operators

Allowing up to one word between terms:

- N1 (Cinahl)
- Adj2 (Medline)
- Near/1 (Web of Science)
- Pre/1 (Scopus)

Allowing up to two words between terms:

- N2 (Cinahl)
- Adj3 (Medline)
- Near/2 (Web of Science)
- Pre/2 (Scopus)

Medline

Ovid MEDLINE(R) ALL <1946 to April 26, 2024>

| **#** | **Searches** | **Results** |
| --- | --- | --- |
| 1 | exp Telemedicine/ or Patient Portals/ or Computers, Handheld/ or Smartphone/ or Mobile Applications/ or Digital Health/ or exp Telemetry/ | 81863 |
| 2 | (mobile health* or mhealth* or m health* or telehealth* or tele health* or ehealth* or e-health* or digital health*).ti,ab,kf. | 48106 |
| 3 | (telemedic* or tele medic* or telenursing or tele nursing or telemonitor* or tele monitor* or telemetr* or (remote adj2 monitor*) or telecare or tele care or teleconsult* or tele consult* or distance consult* or distant consult* or remote consult* or telecounsel* or tele counsel* or distance counsel* or remote counsel* or econsult* or e-consult* or e-coach*).ti,ab,kf. | 49352 |
| 4 | (mobile technolog* or health technolog* or patient portal* or app or apps or phone application* or telephone application* or mobile application* or health application* or smart application* or mhapps or gaming or gamification or exergam* or serious gam*).ti,ab,kf. | 79265 |
| 5 | (smartphone* or ipad or ipads or mobile device* or handheld computer* or tablet computer* or iphone* or android or smartwatch* or smart watch* or wearable* or interactive voice response*).ti,ab,kf. | 67519 |
| 6 | or/1-5 | 229193 |
| 7 | exp Heart Failure/ or ((heart* or cardiac or cardial or myocardial) adj3 (failure* or decompensat* or insufficienc* or incompetenc* or standstill or stand-still)).mp. | 285667 |
| 8 | exp "diet, food, and nutrition"/ or exp micronutrients/ or exp feeding behavior/ or exp Drinking Behavior/ or exp body composition/ or Body Weight/ or body weight changes/ or exp weight gain/ or exp weight loss/ or exp overweight/ or exp thinness/ or exp Sodium, dietary/ or beta vulgaris/ or dh.fs. | 2588417 |
| 9 | (diet* or eat or eating or feed* or food or meal or meals or portion size* or fasting or nutrition* or nutrient* or micronutrient* or macronutrient* or salt or sodium chloride or beet* or beta vulgaris or calory or calori* or drink* or beverage* or ((fluid* or energy) adj2 intake) or (alcohol adj2 consumption) or weight or body composition or overweight or thin* or obes* or adipos* or cachexia).mp. | 4565895 |
| 10 | 8 or 9 | 5528559 |
| 11 | 6 and 7 and 10 | 586 |
| 12 | 11 not (exp Animals/ not exp Humans/) | 559 |
| 13 | limit 12 to yr="2014 -Current" | 415 |

<https://ovidsp.ovid.com/ovidweb.cgi?T=JS&NEWS=N&PAGE=main&SHAREDSEARCHID=6h8cYSABVPRewtwmLBOweVQV3Ga5XuTN3DlVbGFh5Wi3n2BnrKjMZPH9hn1R9WzFk>

Cinahl

Interface - EBSCOhost Research Databases 
Search Screen - Advanced Search 
Database - CINAHL with Full Text

Search modes - Boolean/Phrase

| **#** | **Query** | | **Results** |
| --- | --- | --- | --- |
| S5 | S4 NOT ((MH "Animals+" OR MH "Mammals+" OR MH "Animal Studies") NOT MH "Human") | Limiters - Publication Date: 20140101-20241231 | 160 |
| S4 | S1 and S2 and S3 | | 245 |
| S3 | (MH "Nutrition+") or (MH "Eating Behavior+") or (MH "Drinking Behavior+") or (MH "Nutrients+") or (MH "Beet") or (MH "Sodium, Dietary+") or (MH "Body Weight") or (MH "Body Weight Changes+") or (MH "Body Composition+") or diet* or eat or eating or feed* or food or meal or meals or portion size* or fasting or nutrition* or nutrient* or micronutrient* or macronutrient* or salt or sodium chloride or beet* or beta vulgaris or calory or calori* or drink* or beverage* or ((fluid* or energy) N1 intake) or (alcohol N1 consumption) or weight or body composition or overweight or thin* or obes* or adipos* or cachexia | | 1,053,523 |
| S2 | (MH "Heart Failure+") or ((heart* or cardiac or cardial or myocardial) N2 (failure* or decompensat* or insufficienc* or incompetenc* or standstill or stand-still)) | | 81,833 |
| S1 | (MH "Telehealth+") or (MH "Wearable Sensors") or (MH "Digital Health") or (MH "Patient Portals") or (MH "Mobile Applications") or (MH "Computers, Portable") OR (MH "Computers, Hand-Held") OR (MH "Smartphone") or (MH "Telemetry") or mobile health* or mhealth* or m health* or telehealth* or tele health* or ehealth* or e-health* or digital health* or telemedic* or tele medic* or telenursing or tele nursing or telemonitor* or tele monitor* or telemetr* or (remote N1 monitor*) or telecare or tele care or teleconsult* or tele consult* or distance consult* or distant consult* or remote consult* or telecounsel* or tele counsel* or distance counsel* or remote counsel* or econsult* or e-consult* or e-coach* or mobile technolog* or health technolog* or patient portal* or app or apps or phone application* or telephone application* or mobile application* or health application* or smart application* or mhapps or gaming or gamification or exergam* or serious gam* or smartphone* or ipad or ipads or mobile device* or handheld computer* or tablet computer* or iphone* or android or smartwatch* or smart watch* or wearable* or interactive voice response* | | 103,673 |

Web of Science

| # | Search Query | Results |
| --- | --- | --- |
| 1 | **TI**=(mobile-health* or mhealth* or m-health* or telehealth* or tele-health* or ehealth* or e-health* or digital-health* or telemedic* or tele-medic* or telenursing or tele-nursing or telemonitor* or tele-monitor* or telemetr* or (remote near/1 monitor*) or telecare or tele-care or teleconsult* or tele-consult* or distance-consult* or distant-consult* or remote-consult* or telecounsel* or tele-counsel* or distance-counsel* or remote-counsel* or econsult* or e-consult* or e-coach* or mobile-technolog* or health-technolog* or patient-portal* or app or apps or phone-application* or telephone-application* or mobile-application* or health-application* or smart-application* or mhapps* or gaming or gamification or exergam* or serious-gam* or smartphone* or ipad or ipads or mobile-device* or handheld-computer* or tablet-computer* or iphone* or android or smartwatch* or smart-watch* or wearable* or interactive-voice-response*) or **AB**=(mobile-health* or mhealth* or m-health* or telehealth* or tele-health* or ehealth* or e-health* or digital-health* or telemedic* or tele-medic* or telenursing or tele-nursing or telemonitor* or tele-monitor* or telemetr* or (remote near/1 monitor*) or telecare or tele-care or teleconsult* or tele-consult* or distance-consult* or distant-consult* or remote-consult* or telecounsel* or tele-counsel* or distance-counsel* or remote-counsel* or econsult* or e-consult* or e-coach* or mobile-technolog* or health-technolog* or patient-portal* or app or apps or phone-application* or telephone-application* or mobile-application* or health-application* or smart-application* or mhapps* or gaming or gamification or exergam* or serious-gam* or smartphone* or ipad or ipads or mobile-device* or handheld-computer* or tablet-computer* or iphone* or android or smartwatch* or smart-watch* or wearable* or interactive-voice-response*) or **AK**=(mobile-health* or mhealth* or m-health* or telehealth* or tele-health* or ehealth* or e-health* or digital-health* or telemedic* or tele-medic* or telenursing or tele-nursing or telemonitor* or tele-monitor* or telemetr* or (remote near/1 monitor*) or telecare or tele-care or teleconsult* or tele-consult* or distance-consult* or distant-consult* or remote-consult* or telecounsel* or tele-counsel* or distance-counsel* or remote-counsel* or econsult* or e-consult* or e-coach* or mobile-technolog* or health-technolog* or patient-portal* or app or apps or phone-application* or telephone-application* or mobile-application* or health-application* or smart-application* or mhapps* or gaming or gamification or exergam* or serious-gam* or smartphone* or ipad or ipads or mobile-device* or handheld-computer* or tablet-computer* or iphone* or android or smartwatch* or smart-watch* or wearable* or interactive-voice-response*) | 510350 |
| 2 | **TI**=((heart* OR cardiac OR cardial OR myocardial) NEAR/2 (failure* OR decompensat* OR insufficienc* OR incompetenc* OR standstill OR stand-still)) or **AB**=((heart* OR cardiac OR cardial OR myocardial) NEAR/2 (failure* OR decompensat* OR insufficienc* OR incompetenc* OR standstill OR stand-still)) or **AK**=((heart* OR cardiac OR cardial OR myocardial) NEAR/2 (failure* OR decompensat* OR insufficienc* OR incompetenc* OR standstill OR stand-still)) | 262684 |
| 3 | **TI**=(diet* or eat or eating or feed* or food or meal or meals or portion-size* or fasting or nutrition* or nutrient* or micronutrient* or macronutrient* or salt or sodium-chloride or beet* or beta-vulgaris or calory or calori* or drink* or beverage* or ((fluid* or energy) NEAR/1 intake) or (alcohol NEAR/1 consumption) or weight or body-composition or overweight or thin* or obes* or adipos* or cachexia) or **AB**=(diet* or eat or eating or feed* or food or meal or meals or portion-size* or fasting or nutrition* or nutrient* or micronutrient* or macronutrient* or salt or sodium-chloride or beet* or beta-vulgaris or calory or calori* or drink* or beverage* or ((fluid* or energy) NEAR/1 intake) or (alcohol NEAR/1 consumption) or weight or body-composition or overweight or thin* or obes* or adipos* or cachexia) or **AK**=(diet* or eat or eating or feed* or food or meal or meals or portion-size* or fasting or nutrition* or nutrient* or micronutrient* or macronutrient* or salt or sodium-chloride or beet* or beta-vulgaris or calory or calori* or drink* or beverage* or ((fluid* or energy) NEAR/1 intake) or (alcohol NEAR/1 consumption) or weight or body-composition or overweight or thin* or obes* or adipos* or cachexia) | 7624826 |
| 4 | #3 AND #2 AND #1 AND PY=2014-2024 | 391 |

<https://www.webofscience.com/wos/woscc/summary/a7179afc-f0ca-4d05-9fe4-22eeae6decfa-e3e5f11b/relevance/1>

Scopus

Advanced query

( **TITLE-ABS-KEY** ( diet* OR eat OR eating OR feed* OR food OR meal OR meals OR portion-size* OR fasting OR nutrition* OR nutrient* OR micronutrient* OR macronutrient* OR salt OR sodium-chloride OR beet* OR beta-vulgaris OR calory OR calori* OR drink* OR beverage* OR ( ( fluid* OR energy ) W/1 intake ) OR ( alcohol W/1 consumption ) OR weight OR body-composition OR overweight OR thin* OR obes* OR adipos* OR cachexia ) ) AND ( **TITLE-ABS-KEY** ( ( heart* OR cardiac OR cardial OR myocardial ) W/2 ( failure* OR decompensat* OR insufficienc* OR incompetenc* OR standstill OR stand-still ) ) ) AND ( **TITLE-ABS-KEY** ( mobile-health* OR mhealth* OR m-health* OR telehealth* OR tele-health* OR ehealth* OR e-health* OR digital-health* OR telemedic* OR tele-medic* OR telenursing OR tele-nursing OR telemonitor* OR tele-monitor* OR telemetr* OR ( remote W/1 monitor* ) OR telecare OR tele-care OR teleconsult* OR tele-consult* OR distance-consult* OR distant-consult* OR remote-consult* OR telecounsel* OR tele-counsel* OR distance-counsel* OR remote-counsel* OR econsult* OR e-consult* OR e-coach* OR mobile-technolog* OR health-technolog* OR patient-portal* OR app OR apps OR phone-application* OR telephone-application* OR mobile-application* OR health-application* OR smart-application* OR mhapps* OR gaming OR gamification OR exergam* OR serious-gam* OR smartphone* OR ipad OR ipads OR mobile-device* OR handheld-computer* OR tablet-computer* OR iphone* OR android OR smartwatch* OR smart-watch* OR wearable* OR interactive-voice-response* ) ) AND PUBYEAR > 2013 AND NOT INDEXTERMS ( ( animal* OR nonhuman ) AND NOT human* )

1012 results
